# Electrocardiographic manifestations in female team handball players: analyzing ECG changes in athletes

**DOI:** 10.3389/fspor.2024.1384483

**Published:** 2024-04-26

**Authors:** A. Malmgren, E. Trägårdh, P. Gudmundsson, B. Kjellström, M. Stagmo, M. Dencker

**Affiliations:** ^1^ Department of Translational Medicine, Lund University, Malmö, Sweden; ^2^ Department of Medical Imaging and Physiology, Skåne University Hospital, Malmö, Sweden; ^3^ Department of Biomedical Science, Faculty of Health and Society, Malmö University, Malmö, Sweden; ^4^ Department of Clinical Sciences Lund, Lund University, Clinical Physiology and Skåne University Hospital, Lund, Sweden; ^5^ Department of Cardiology, Skåne University Hospital, Malmö, Sweden

**Keywords:** athlete, echocardiography, electrocardiography, left ventricle, left ventricular mass

## Abstract

**Introduction:**

Long-term intense training leads to structural, functional, and electrical remodeling of the heart. How different sports affect the heart has not been fully investigated, particularly for female athletes. The aim of the present study was to investigate the morphology of 12-lead resting electrocardiogram (ECG) in elite female handball players compared to non-athlete female subjects. Potential changes will be explored to see if they could be explained by differences in cardiac dimensions and exercise hours.

**Materials and methods:**

A cross-sectional study of 33 elite female team handball players compared to 33 sex and age-matched, non-athletic controls (age range 18–26 years) was performed. All participants underwent a resting 12-lead ECG and an echocardiographic examination. ECG variables for left ventricular hypertrophy and durations were evaluated and adjusted for cardiac dimensions and exercise hours using ANCOVA analysis. A linear regression analysis was used to describe relation between echocardiographic and ECG measures and exercise hours.

**Results:**

The female handball players had larger cardiac dimensions and significantly lower heart rate and QTc duration (Bazett's formula) as well as increased QRS and QT durations compared to controls. The 12-lead sum of voltage and the 12-lead sum of voltage ∗ QRS were significantly higher among handball players. Changes in ECG variables reflecting the left ventricle could in part be explained by left ventricular size and exercise hours. Correlation with exercise hours were moderately strong in most of the echocardiographic measures reflecting left ventricular (LV), left ventricular mass (LVM), left atrium (LA) and right atrium (RA) size. Poor to fair correlations were seen in the majority of ECG measures.

**Conclusions:**

Female team handball players had altered ECGs, longer QRS and QT durations, higher 12-lead sum of voltage and 12-lead sum of voltage ∗ QRS as well as shorter QTc (Bazett's formula) duration compared to non-athletic controls. These findings could only partly be explained by differences in left ventricular size. Despite larger atrial size in the athletes, no differences in *P*-wave amplitude and duration were found on ECG. This suggest that both structural, and to some degree electrical remodeling, occur in the female team handball players' heart and highlight that a normal ECG does not rule out structural adaptations. The present study adds knowledge to the field of sports cardiology regarding how the heart in female team handball players adapts to this type of sport.

## Introduction

Regular and long-term intense physical exercise leads to structural, functional, and electrical remodeling of the heart. These physiological changes affect the atrial and ventricular dimensions and are known as the athlete's heart and can be observed in both men and women (1–3). The degree of morphological changes depend on factors such as age, body size, ethnicity, sex, and sport activity and intensity (4–7).

A 12-lead electrocardiogram (ECG) in combination with a physical examination and medical history is recommended by the European Society of Cardiology as a pre-participation screening protocol of athletes to identify cardiac abnormalities that may reflect an underlying heart disease (8). ECG changes in athletes are common and international recommendations for ECG interpretation were recently updated to help clinicians differentiate between physiological and pathological ECG changes to decide if further investigation is warranted (9).

Even though the physiological aspects of the athlete's heart are well-known, ECG data from female athletes in specific sports is scarce. Sports are classified based on degree of dynamic and static components during competition and training (10). Team handball exercise have high dynamic and moderate static components leading to high maximal O_2_ uptake, increased cardiac output and, to some degree, increased blood pressure load (10). The aim of the present study was to investigate potential training-induced changes in a resting 12-lead ECG among female team handball players compared to healthy, non-athletic female subjects. In addition, we aimed to explore whether the changes could be explained by differences in cardiac dimensions, left ventricular mass (LVM) and duration in exercise.

## Materials and methods

### Study population

This cross-sectional study included 35 elite female handball players (athletes) and 33 non-athletic (controls) females matched for age, all ≥18 years. The athletes were recruited locally out of three highly ranked teams participating in the Swedish national league and were training >4 h/week. Controls were Malmö university students who volunteered after an oral presentation of the study and performed no organized physical activity/week, e.g., cycling or walking. All participants completed a questionnaire to assess training time, type of training and to rule out any known history of cardiovascular disease. Exclusion criteria were <18 years old, cardiovascular disease, cease in training or injuries among athletes or more than two hours organized physical activity/week among control subjects.

Of the 35 athletes, two were excluded because of cardiovascular disease; one had a bicuspid aortic valve and one due to recent hospitalization for suspected myocarditis. Thus, the final study population consisted of 33 athletes and 33 controls.

The study was approved by the Swedish Ethical Review Authority in Lund (Dnr 2012/77) and was conducted in accordance with the principles set forth in the Helsinki Declaration. All participants gave their written, informed consent to participate.

### Electrocardiogram

**Figure 1:**
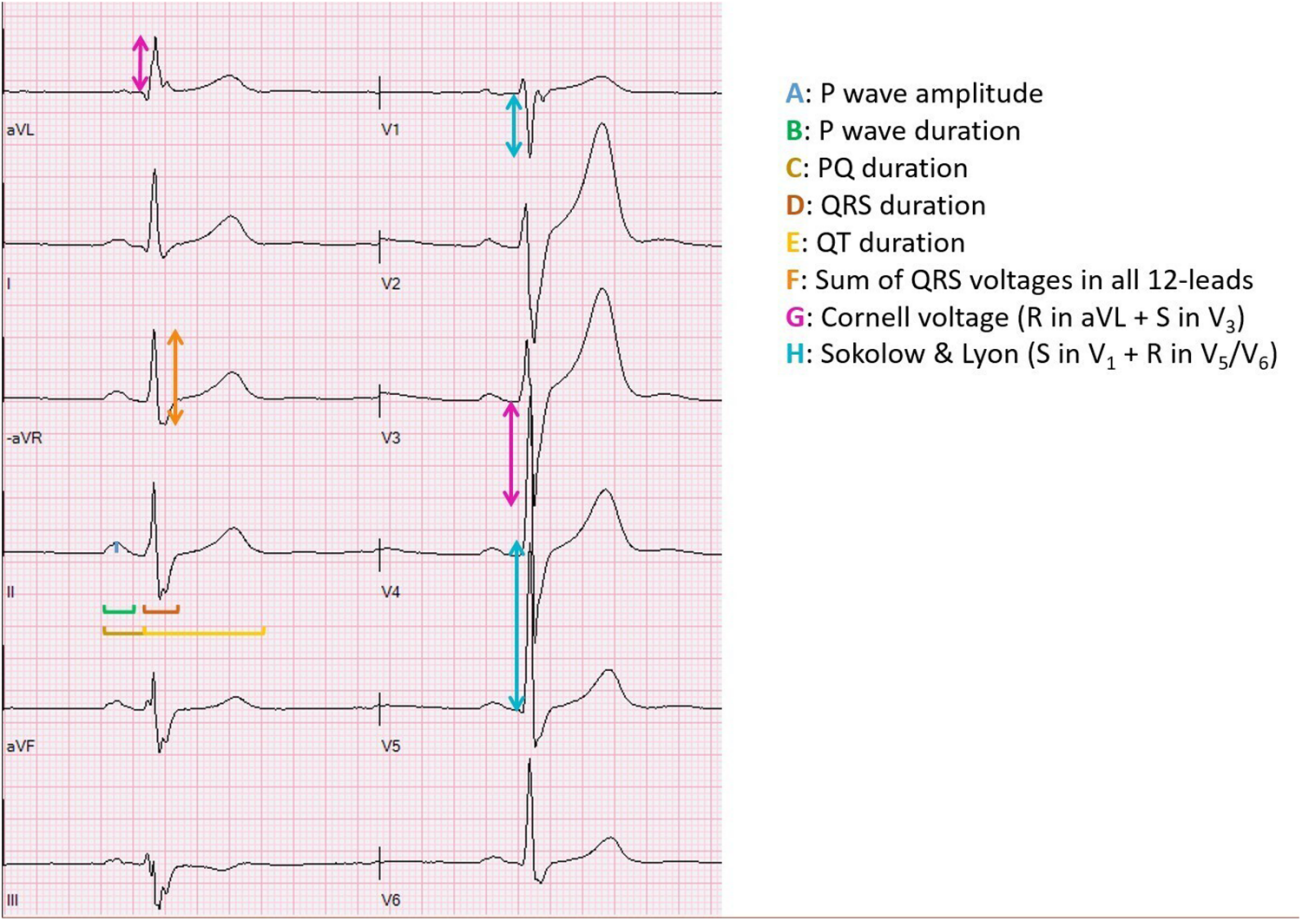
Illustration of the ECG measurements. This illustration was made by Andreas Malmgren, the first author of the manuscript.

A 12-lead resting ECG at paper speed 50 mm/s was recorded using GE Marquette MAC 5,000 resting ECG system (GE Medical systems, Milwaukee, WI, USA). Prior to ECG recording, all participants rested quietly in a dimmed lighting room for 10 min in supine position. Good technical quality of the ECG was assured by clear ECG signals in all leads, no muscle tremor artifacts or electromagnetic interference and good electrode contact. The following ECG variables were measured/noted and are illustrated in Figure 1:

• Heart rate (HR)
• *P*-wave duration
• *P*-wave amplitude
• PQ duration
• *R*-wave axis
• QRS duration
• QT duration and corrected for heart rate according to Bazett's formula: $QTcB=QT÷\sqrt{\left(R-R\right)}$ and according to Fridericia formula: $QTcF=QT÷(R-R)\frac{1}{3}$
• Left ventricular dimension/size (six different measurements): (1) The sum of QRS voltage in all 12-leads, from the peak of the *R* wave to the nadir of either the *Q* wave or the *S* wave, whichever was deeper, (2) Cornell voltage (amplitude of *R* in aVL + amplitude of *S* in V_3_ (11), (3) Sokolow & Lyon (amplitude of *S* in V_1_ + amplitude of *R* in V_5_ or V_6_) (12), (4) A voltage-duration product for each of the three measurements was calculated by multiplying the voltage criterion by QRS duration
• Presence of incomplete right bundle branch block (RBBB) (an rSR' complex in precordial leads V1-V2 and QRS duration <120 ms)
• Presence of early repolarization (concave ST-segment elevation in precordial leads)

Sinus bradycardia was defined as HR below 50 beats/min and short PQ was defined with duration <120 ms. Left atrial (LA) enlargement was defined as prolonged *P* wave duration >120 ms in lead II with negative portion of the *P* wave ≥0.1 mV in depth and right atrial (RA) enlargement was defined as ≥0.25 mV in depth in lead II. All ECG data were generated from software MUSE™ NX (GE HealthCare, version 10.1.5.200087, General Electrics, Boston, MA, USA).

### Echocardiography

The echocardiographic examination has been described in detail previously (13). In summary, echocardiographic examinations and measurements of left ventricular (LV), LA and RA size, and calculation of LVM were performed by one experienced echocardiographer according to current recommendations using a Philips iE33 ultrasound system (Koninklijke Philips N.V.) equipped with S5-1 and X5-1 transducers. Echocardiographic measurements of left ventricular (LV) and left atrial (LA) size, and calculation of LVM were performed according to current recommendations (14). All cardiac dimensions were indexed for body surface area (BSA).

### Statistical analysis

All analyses were performed using Statistical Package for the Social Sciences (IBM, SPSS Statistics, and version 25, Chicago, IL, USA) and in consultation with a professional statistician. A power calculation to determine sample size needed and with consideration to achievability resulted in approximately 30 female handball players and equally in corresponding non-athletic control group. Given alfa 0.05 and beta 0.10, the size of the present study is sufficiently with 95% possibility to detect differences between groups with 1 standard deviation which we consider to be a relevant difference. Continuous data were expressed as mean ± standard deviation (SD) and with 95% confidence interval (CI). Categorical data were expressed in absolute numbers. Normal distribution was assessed visually from histograms. The independent Student's *t*-test was used to test for between-group mean differences in subject characteristics, ECG and echocardiographic measures. Analysis of covariance (ANCOVA) tests were used to adjust ECG measures where statistical differences between the two groups were seen for the influence of cardiac dimensions measured by echocardiography and for exercise hours. A linear regression analysis was performed to describe the relation between echocardiographic and ECG measures (dependent variables) compared to exercise hours (independent variable). Pearson's correlation coefficient (*r*-values) was used as appropriate. Degree of correlation between tests was classified as either poor (*r* = less than 0.3), fair (*r* = 0.3–0.5), moderately strong (*r* = 0.6–0.8) and very strong (*r* = at least 0.8) (15). A *p*-value < 0.05 was considered statistically significant.

## Results

The athletes were younger than controls (Table 1) and trained 10.8 ± 2.3 (range 5–17.5) hours per week compared to the controls who performed no more than 2 h per week. Athletes' training included on average 1.7 h of strength training and 1.8 h of fitness training, e.g., core training and preventively for injuries. The athletes had significantly larger cardiac dimensions than controls, see Table 1.

**Table 1:** Subject characteristics and echocardiographic findings expressed as mean ± SD using student's *t*-test. Echocardiographic measurements are adjusted for body surface area.

	Handball players	Non-athletic controls	*p*	95% CI
	(*N* = 33)	(*N* = 33)		
Age (years)	20.0 ± 2.0	23.0 ± 2.0	<0.001	−3.7–−1.9
Height (cm)	175.0 ± 6.9	170.9 ± 5.5	0.01	1.0–7.2
Body Mass (kg)	73.2 ± 7.9	64.3 ± 8.7	<0.001	4.8–13.0
BMI (kg/m^2^)	23.9 ± 2.0	22.0 ± 2.9	0.003	0.7–3.1
BSA (m^2^)	1.88 ± 0.13	1.75 ± 0.12	<0.001	0.07–0.19
LAd (mm/m^2^)	19.3 ± 1.5	18.4 ± 1.6	0.03	0.1–1.6
LAV 2DE (ml/m^2^)	34.1 ± 6.2	24.6 ± 4.1	<0.001	6.9–12.0
LAV 3DE (ml/m^2^)	29.4 ± 5.8	22.2 ± 3.6	<0.001	4.8–9.5
LVIDd (mm/m^2^)	27.1 ± 1.7	26.3 ± 1.8	0.05	−0.01–1.7
LVEDV 2DE (ml/m^2^)	68.4 ± 7.3	52.9 ± 5.6	<0.001	12.4–18.7
LVEDV 3DE (ml/m^2^)	69.4 ± 8.2	55.0 ± 6.8	<0.001	10.7–18.1
LVM (g/m^2^)	73.5 ± 12.3	55.7 ± 7.1	<0.001	12.9–22.8
RAa (cm^2^/m^2^)	9.5 ± 1.1	7.8 ± 0.9	<0.001	1.1–2.1

BMI, body mass index; BSA, body surface area; cm, centimeter; CI, confidence interval; g, gram; kg, kilogram; LAd, left atrium diameter in systole measured by 2-dimensional echocardiography; LAV 2DE, left atrium volume in end-systole measured by 2-dimensional echocardiography; LAV 3DE, left atrium volume in end-systole measured by 3-dimensional echocardiography; LVEDV 2DE, left ventricular end-diastolic volume measured by 2-dimensional echocardiography; LVEDV 3DE, left ventricular end-diastolic volume measured by 3-dimensional echocardiography; LVIDd, left ventricular internal diameter in end-diastole measured by 2-dimensional echocardiography; LVM, left ventricular mass; RAa, right atrium area in systole; m, meter; ml, milliliter; mm, millimeter; SD, standard deviation.

CI is showing that the difference in the studied population lies between the lowest and highest limits with 95% confidence.

All participants were in sinus rhythm and most of the ECG measurements were within normal limits. Nine controls and two athletes had short PQ duration and low voltage was found in one control. The 11 individuals with short PQ duration and one also with low voltage had no delta wave on the ECG which according to standard clinical evaluation, in addition to normal echocardiographic examination suggests normal findings.

Sinus bradycardia and early repolarization were more common among athletes than controls and the athletes had lower HR and QTc duration according to Bazett's formula. QTc duration according to Fridericia showed no difference between groups. Furthermore, the athletes had longer QRS and QT durations, see Table 2. The 12-lead sum of voltage and 12-lead voltage-duration product were higher in athletes than controls. Measurements of left ventricular size using the Sokolow & Lyon and Cornell criterion showed no difference between groups.

**Table 2:** Electrocardiographic findings expressed as mean ± SD using student's *t*-test or as number.

	Handball players (*N* = 33)	Non-athletic controls (*N* = 33)	*p*	95% CI
Heart rate (beats/min)	53 ± 8	67 ± 12	<0.001	−19.3–−9.1
*P* wave duration (ms)	98 ± 10	96 ± 13	0.448	−3.5–7.9
*P* wave amplitude (mV)	0.10 ± 0.04	0.11 ± 0.05	0.327	−0.03–0.01
PQ duration (ms)	150 ± 20	141 ± 21	0.097	−1.6–18.8
QRS duration (ms)	91 ± 9	86 ± 7	0.026	0.6–8.7
12-lead sum of voltage (mV)	15.5 ± 2.4	14.1 ± 2.6	0.029	0.15–2.6
12-lead sum of voltage (mV) ∗ QRS	1,413 ± 261	1,226 ± 267	0.005	57.6–317.1
Cornell voltage (mV)	0.88 ± 0.36	0.81 ± 0.39	0.49	−0.12–0.25
Cornell voltage (mV) ∗ QRS	80 ± 33	72 ± 37	0.349	−9.2–25.6
Sokolow & Lyon (mV)	2.3 ± 0.6	2.2 ± 0.6	0.631	−0.2–0.3
Sokolow & Lyon (mV) ∗ QRS	206 ± 51	192 ± 54	0.276	−11.6–40.1
R axis	73 ± 14	63 ± 19	0.02	1.6–18.4
QT duration (ms)	436 ± 29	399 ± 31	<0.001	22.1–51.7
QTc duration (ms) Bazett's	406 ± 21	417 ± 20	0.024	−21.4–−1.5
QTc duration (ms) Fridericia	415 ± 17	411 ± 17	0.324	−4.3–12.7
Bradycardia (*n*)	9	2		
Early repolarization (*n*)	3	0		
Right axis deviation (*n*)	2	0		
Incomplete RBBB (*n*)	1	0		
Short PQ duration (*n*)	2	9		
Low voltage	0	1		

CI, confidence interval; ms, millisecond; mV, millivolt; min, minute; n, number; RBBB, right bundle branch block; SD, standard deviation.

CI is showing that the difference in the studied population lies between the lowest and highest limits with 95% confidence.

Athletes had higher QRS duration, 12-lead sum of voltage and 12-lead sum of voltage product adjusted for LV internal diameter in diastole (LVIDd) as well as higher QT duration after adjustment for LVM and for LVIDd compared to controls which is presented in Table 3. When ECG measurements were adjusted for exercise hours, the 12-lead sum of voltage was higher in athletes compared to controls, see Table 4.

**Table 3:** ECG variables adjusted for echocardiographic measurements using ANCOVA test.

	LVIDd (mm/m^2^)		LVEDV 2DE (ml/m^2^)		LVEDV 3DE (ml/m^2^)		LVM (g/m^2^)	
	β (95% CI)	*p*	β (95% CI)	*p*	β (95% CI)	*p*	β (95% CI)	*p*
QRS duration (ms)	4.9 (0.7–9.2)	0.02	−0.8 (−7.0–5.5)	0.81	1.5 (−4.1–7.1)	0.6	4.6 (−0.9–10.2)	0.10
12-lead sum of voltage (mV)	1.46 (0.19–2.72)	0.027	0.63 (−1.29–2.55)	0.52	−0.05 (−1.69–1.58)	0.95	1.38 (−0.28–3.03)	0.11
12-lead sum of voltage (mV) ∗ QRS	194 (61.8–326)	0.006	34.9 (−161–231)	0.73	10.1 (−157–178)	0.91	174 (1.84–347)	0.05
QT duration (ms)	36.3 (20.9–51.7)	<0.001	9.8 (−12.0–31.6)	0.37	15.7 (−3.7–35.1)	0.11	37.1 (17.0–57.2)	<0.001
QTc duration (ms) Bazett's formula	−9.1 (−19.1–0.9)	0.07	−8.9 (−24.6–6.8)	0.26	−9.2 (−23.1–4.7)	0.19	−10.4 (−23.9–3.0)	0.13

CI, confidence interval; g, gram; LAd, left atrium diameter in systole; LAV 2DE, left atrium volume in end-systole measured by 2-dimensional echocardiography; LAV 3DE, left atrium volume in end-systole measured by 3-dimensional echocardiography; LVEDV 2DE, left ventricular end-diastolic volume measured by 2-dimensional echocardiography; LVEDV 3DE, left ventricular end-diastolic volume measured by 3-dimensional echocardiography; LVIDd, left ventricular internal diameter in end-diastole; LVM, left ventricular mass; m, meter; mm, millimeter; ms, millisecond; mV, millivolt.

A positive β mean that the handball players have higher values compared to controls and a negative β mean that handball players have lower values compared to controls.

CI is showing that the difference in the studied population lies between the lowest and highest limits with 95% confidence.

**Table 4:** ECG variables adjusted for exercise hours using ANCOVA test.

	Adjusted for exercise hours	
	β (95% CI)	*p*
QRS duration (ms)	−8.0 (−21.0–5.1)	0.23
12-lead sum of voltage (mV)	5.46 (1.57–9.36)	0.01
12-lead sum of voltage (mV) ∗ QRS	370 (−49.1–788)	0.09
QT duration (ms)	44.5 (−4.6–93.5)	0.08
QTc duration (ms)	4.9 (−27.7–37.4)	0.77

CI, confidence interval; ms, millisecond; mV, millivolt.

A positive β mean that the handball players have higher values compared to controls and a negative β mean that handball players have lower values compared to controls.

CI is showing that the difference in the studied population lies between the lowest and highest limits with 95% confidence.

Correlation with exercise hours was moderately strong for HR (*r* = 0.551), LAV 2DE (*r* = 0.671), LAV 3DE (*r* = 0.607), LVEDV 2DE (*r* = 0.764), LVEDV 3DE (*r* = 0.668), LVM (*r* = 0.653) and RAa (*r* = 0.597). A fair correlation with exercise hours was seen with QRS duration (*r* = 0.334), R axis (*r* = 0.302), QT duration (*r* = 0.492), QTcB (*r* = 0.303), LAd (*r* = 0.305). A poor correlation with exercise hours was seen with *P* wave duration (*r* = 0.077), *P* wave amplitude (*r* = 0.130), PQ duration (*r* = 0.232), 12-lead sum of voltage (*r* = 0.180), 12-lead sum of voltage ∗ QRS (*r* = 0.291), Cornell voltage (*r* = 0.075), Cornell voltage ∗ QRS (*r* = 0.119), Sokolow & Lyon (*r* = 0.042), Sokolow & Lyon ∗ QRS (*r* = 0.057), QTcF (*r* = 0.075), LVIDd (*r* = 0.204). All data are shown in Table 5.

**Table 5:** Linear regression analysis with exercise hours used as independent variable.

Dependent variables	*r*	*r* square	*p*
Heart rate (beats/min)	0.551	0.304	<0.001
*P* wave duration (ms)	0.077	0.006	0.537
*P* wave amplitude (mV)	0.130	0.017	0.297
PQ duration (ms)	0.232	0.054	0.061
QRS duration (ms)	0.334	0.112	0.006
12-lead sum of voltage (mV)	0.180	0.032	0.148
12-lead sum of voltage (mV) ∗ QRS	0.291	0.085	0.018
Cornell voltage (mV)	0.075	0.006	0.549
Cornell voltage (mV) ∗ QRS	0.119	0.014	0.343
Sokolow & Lyon (mV)	0.042	0.002	0.737
Sokolow & Lyon (mV) ∗ QRS	0.057	0.003	0.648
R axis	0.302	0.091	0.014
QT duration (ms)	0.492	0.242	<0.001
QTc duration (ms) Bazett's	0.303	0.092	0.014
QTc duration (ms) Fridericia	0.075	0.006	0.547
LAd (mm/m^2^)	0.305	0.093	0.013
LAV 2DE (ml/m^2^)	0.671	0.450	<0.001
LAV 3DE (ml/m^2^)	0.607	0.369	<0.001
LVIDd (mm/m^2^)	0.204	0.042	0.100
LVEDV 2DE (ml/m^2^)	0.764	0.584	<0.001
LVEDV 3DE (ml/m^2^)	0.668	0.447	<0.001
LVM (g/m^2^)	0.653	0.427	<0.001
RAa (cm^2^/m^2^)	0.597	0.356	<0.001

m, meter; mm, millimeter; ml, milliliter; ms, millisecond; mV, millivolt; min, minute; cm, centimeter; g, gram; kg, kilogram; LAd, left atrium diameter in systole measured by 2-dimensional echocardiography; LAV 2DE, left atrium volume in end-systole measured by 2-dimensional echocardiography; LAV 3DE, left atrium volume in end-systole measured by 3-dimensional echocardiography; LVEDV 2DE, left ventricular end-diastolic volume measured by 2-dimensional echocardiography; LVEDV 3DE, left ventricular end-diastolic volume measured by 3-dimensional echocardiography; LVIDd, left ventricular internal diameter in end-diastole measured by 2-dimensional echocardiography; LVM, left ventricular mass; RAa, right atrium area in systole.

## Discussion

The main finding in the present study was that, although most ECG parameters remained within normal limits in all subjects, the female handball players had ECG morphology changes that was not seen in the non-athletic control group. A large cohort study by Pelliccia et al. including both sexes and different sport disciplines showed that 60% of all athletes had normal or minor alterations on ECG and male athletes most commonly showed abnormal ECG patterns (16). The major determinant of altered ECG pattern was morphological cardiac remodeling which was seen in athletes engaged in endurance sports e.g., cycling, canoeing/rowing and cross-country skiing who had the greatest increase in LV size, wall thickness and mass (16). Although the athletes in the present study demonstrated larger cardiac dimensions on echocardiography only ECG changes reflecting LV could partly be explained by differences in LV size. To the best of our knowledge, this is the first study demonstrating to which degree ECG changes could be explained by differences cardiac size in female handball players.

None of the echocardiographic measurements of RA area and LA diameter nor volume influenced ECG parameters reflecting the LA and RA electrical activity. This may indicate that the *P*-wave amplitude and duration reflects LA and RA size poorly and that electrical remodeling is, in part, independent of atrial size. Similar findings have been observed previously in a longitudinal study in young healthy team sports athletes who had training-induced biatrial enlargement without any association to *P*-wave morphology (17). Previously studies by Lee et al. and Tsao et al. have described that abnormalities in *P*-wave morphology on ECG more likely indicate nonspecific LA or RA abnormalities rather than atrial enlargement (18, 19).

In contrast to the atrial size, LV volume in the present study has an impact on ECG measurements, and partly this is also the case for LVM. Linear measurement of LVM using M-mode echocardiography assume perpendicular alignment of the ultrasound beam to the LV long axis and is known to be less accurate and reproducible compared with 2D and 3D echocardiography especially in abnormally shaped left ventricles and underestimates LVM (20–22). This may, in part, explain why LVM measured by M-mode echocardiography wasn't able to explain differences seen on ECG in the present study. The measurements of the LV diameter differed somewhat from the measurements of LV volume, and this could be explained by known shortcomings of linear measurements compared to volume measurements when assessing LV size. Echocardiographic 2D and 3D volumes as measures of cardiac size have been shown to have a good correlation with cardiac magnetic resonance imaging (23).

QT duration represents the total time of depolarization and repolarization and prolongation may predispose life-threatening arrhythmias which makes this crucial to assess when interpreting ECG. Despite the significant differences in QT and QTc durations using Bazett's formula seen between the two groups none of the participants had pathological QTc durations ≥480 ms. Although Bazett's formula is recommended and the most used in clinical practice it shows less accuracy at lower HR <60 bpm (24, 25) which is a common finding in athletes. This was the reason why we also corrected the QT time using the Fridericia's formula which may be preferred in athletes screening (26).

In contrast to Bazett, the use of Fridericia showed no differences in QTc duration between the two groups in the present study, confirming that HR in athletes must be considered.

Correct interpretation of ECG in athletes is essential to distinguish between pathological and physiological findings with the aim to identify the athletes who will need further tests. According to current international recommendations, ECG findings like sinus bradycardia, incomplete RBBB, and early repolarizations, as seen in the handball players in the present study, are expected findings (9) and in accordance with other published data in younger trained athletes (27–29). These changes are considered to be related to physiological adaptation to exercise. The studied female handball players' strength and fitness training lead to adaptation of the heart with an increase in cardiac size and wall thickness assessed by echocardiography compared to non-athletic subjects.

None of the study participants showed isolated ECG voltage criteria for LVH using Sokolow & Lyon and Cornell voltage. Since there was no echocardiographic sign of increased left ventricular wall thickness in the present study population this was expected. This finding is in contrast with a previous study including male handball players (25.3 ± 4.4 years) where 19% had ECG signs of LVH according to the Sokolow & Lyon criteria though no increased left ventricular wall thickness was seen in these subjects when assessed with echocardiography (30). Differences in anthropometric variables, cardiac size, fitness, sex and age may all explain this. In a recent study, ECG signs of LVH were less common in women compared to men. It was suggested that lower absolute cardiac dimensions in women might partly explain this finding (31). Furthermore, a recently published study in male athletes showed a weak association between different ECG voltage criteria and LVM, suggesting that the clinical usefulness for ECG in screening for LVH in athletes may be limited (32).

Findings of longer QRS durations, 12-lead sum of voltage and 12-lead voltage-duration product among athletes in the present study suggests that increased LV volumes and LVM may increase the time needed to activate the physiological adapted heart muscle cells in athletes. QRS duration and 12-lead sum of voltage have previously been shown to improve identification of LV hypertrophy (33) and, another study showed a strong correlation between QRS duration and LVM in healthy subjects (34).

Apart from 12-lead sum of voltage, exercised hours affected all other measured ECG variables. The change in 12-lead sum of voltage was borderline significant, and thus, a larger study population could have altered this finding. In addition, questionnaire assessments of physical activity are known to have inaccuracies. With that in mind, the findings in the present study support the theory that longer training duration affects cardiac size and that this may also be reflected as ECG changes. It has previously been shown that 4 h or more of intensive exercise per week is associated with cardiac ECG manifestations (9, 35).

Linear regression analysis demonstrated moderately strong correlation between exercise hours and echocardiographic measures of LV and LA volumes, LVM and RA area. This contrasted with ECG measures where the majority demonstrated poor correlation, however in QRS duration, *R* axis, QT duration and QTcB the correlation was fair. These findings indicate that cardiac remodeling is associated with training load in female handball players as shown by echocardiography but not to the same extent for ECG. Previous studies confirm this finding of exercise hours being a determinant of adaptation in athletes' heart (36–38).

Accurate cardiac imaging assessment and ECG interpretation among athletes is crucial and very important in understanding training-induced physiologic adaptations of the heart. There are numerous types of sports affecting athletes' heart morphology differently due to their dynamic and static components and increased knowledge on how specific training impacts the athlete's heart is needed. The present study report novel findings related to the ECG changes observed in female handball players, a sport combining both components. It would be interesting to perform a longitudinal study combining different modalities e.g., cardiac magnetic resonance, echocardiography and ECG, among handball players of both sexes to evaluate the cardiac effect depending on the position the athletes have on the field, assess how the heart is adapting over time, if long-term training poses any cardiovascular risk and to compare potential differences between sexes.

### Limitations

There are some limitations worth highlighting. First, this is a cross-sectional designed study which makes it difficult to differentiate between cause and effect. The results are based on data collected at rest at one point. Furthermore, the use of this study type cannot estimate any potential risk in playing handball over a longer period of life. Second, a larger study population and with less range in exercise hours among the athletes may be required to find small differences in ECG measurements. Third, it is also worth mentioning the risk of selection bias among participants. The two studied groups are homogenous and may not reflect the heterogeneous society in general. There might be a greater interest in participating in a study like the present if an individual is interested in exercising and health or in getting cardiac examinations as a screening to rule out any unknown pathology. This may have an impact on the generalizability of the present findings to other populations and sports. There could also be a potential risk that subjects in the control group have reported less volume of performed physical activity per week than is actually true to be included in the study according to the inclusion criteria. Unfortunately, we have no information on maximal oxygen uptake (VO_2_) which is another limitation of the present study, since VO_2_ max is known to be a useful value of fitness. Finally, the aim of this study was not to present data to differentiate between normal ECG findings and findings that may indicate pathology. The present study included a rather small study population and only one sport, handball, and the results may not be transferable to other sports. Furthermore, our results are limited to female athletes.

## Conclusions

Female team handball players had altered ECGs, longer QRS and QT durations, higher 12-lead sum of voltage and 12-lead sum of voltage ∗ QRS as well as shorter QTc (Bazett's) duration compared to non-athletic controls. These findings could only partly be explained by differences in left ventricular size. Despite larger atrial size in the athletes, no differences in *P*-wave amplitude and duration were found on ECG. This suggest that both structural and, to some degree, electrical remodeling occur in the female team handball players’ heart and highlight that a normal ECG does not rule out structural adaptations. The present study adds knowledge to the field of sports cardiology regarding how the heart in female team handball players adapts to this type of sport.

## Data Availability

The raw data supporting the conclusions of this article will be made available by the authors, without undue reservation.
